# Biological impact of xeno-free chemically defined cryopreservation medium on amniotic epithelial cells

**DOI:** 10.1186/s13287-015-0258-z

**Published:** 2016-01-12

**Authors:** Toshio Miki, Wisia Wong, Elton Zhou, Anthony Gonzalez, Irving Garcia, Brendan H. Grubbs

**Affiliations:** Department of Biochemistry and Molecular Biology, Keck School of Medicine, University of Southern California, Los Angeles, CA USA; Department of Obstetrics and Gynecology, Keck School of Medicine, University of Southern California, Los Angeles, CA USA; Eli and Edythe Broad Center for Regenerative Medicine and Stem Cell Research at USC, Keck School of Medicine, University of Southern California, Los Angeles, CA USA

**Keywords:** Placenta, Amniotic epithelial cells, Placenta-derived stem cells, Cryopreservation, Serum-free, Xeno-free, Chemically defined media

## Abstract

**Background:**

Amnion-derived stem cells have been proposed for cell replacement therapy and tissue regeneration. An easily accessible cell source, the placenta, allows us to potentially establish a bio-bank of cells for immunotype matched clinical applications. Several xeno-free (XF) cryopreservation media are currently available for pluripotent stem cells, however, these media have not yet been evaluated for the cryopreservation of amnion-derived stem cells.

**Methods:**

Human amniotic epithelial cells were collected using standard protocols, and stored at −160 °C in one of five commercially available media. Cells frozen in standard media containing fetal bovine serum served as controls. Cells were then thawed, and evaluated for viability, mitochondrial membrane stability, and senescence status. Quantitative real time PCR was utilized to assess for expression of stem cell genes, and flow cytometry was used to identify the stem cell surface markers.

**Results:**

Cell recovery and repopulation assays indicated no significant difference between XF media versus standard cryopreservation medium. In addition, no impact was observed on the senescence status, the cytostructural or mitochondrial morphology between the tested cryopreservation media. Differences were observed on the expression of stem cell marker genes (*OCT4, SOX2*, and *NANOG*) and a cell surface marker (TRA1-60) following cryopreservation in different chemically defined XF media, however, these were not statistically significant.

**Conclusions:**

Xeno-free cryopreservation of human amnion-derived stem cells is feasible and can be standardized to establish a bio-bank with human amnion-derived stem cells for future clinical application. Optimization of this media may allow for improved preservation of stem cell-like characteristics.

## Background

Over the last decade, there has been a growing interest in the placenta as a rich source of stem cells [1, 2] without the limitations and ethical concerns associated with the use of other pluripotent stem cell sources. Human amniotic epithelial cells (hAECs) are derived from the epiblast and retain stem cell-like characteristics [3, 4]. Although pluripotency of a single amniotic epithelial (AE) cell has not yet been identified, under appropriate culture conditions hAECs exhibit the capability of differentiating into all three germ layers [5, 6]. A variety of preclinical studies have already shown the promising therapeutic value of hAECs [7–12]. The large volume of available placental tissue in conjunction with limited ethical concerns of the cell source, suggests the possibility of a biobank system to provide immunotype-matched hAECs for future clinical applications [13].

It is essential to establish a reliable and reproducible cryopreservation protocol for a clinically applicable biobanking system. Ideally, the banking system would be networked among multiple procurement centers to provide all needed immunotypes [14]. In order to establish the multicenter network, the cryopreservation protocol needs to be standardized to maintain homogeneity of banked cells, and allow them to maintain their stem cell-like characteristics. This concept has already been applied to the cryopreservation of hematopoietic stem cells for clinical applications [12, 15]. The cryopreservation protocols for other emerging progenitor cell populations have also been demonstrated in several studies [16–19], however, it has not been demonstrated for hAECs.

Conventional cryopreservation media contains fetal bovine serum (FBS), a mixture of growth factors, cytokines and undefined substances which includes bovine exosomes [20–22]. These undefined substances, and the concern for transmission of zoonotic disease, prohibit the use of FBS when establishing a standardized cryopreservation protocol for clinical use in humans. Several serum-free cryopreservation media and methods have been developed and distributed on the market as good manufacturing practice (GMP)-compliant or GMP-amenable products [15, 23–25]. The aim of this study is to demonstrate the feasibility in establishing a standardized cryopreservation protocol with commercially available, xeno-free, chemically defined cryopreservation medium for future clinical therapy.

## Methods

### Isolation and culture of human amniotic epithelial cells

Human placentae were obtained from the Los Angeles County and University of Southern California Medical Center. All placental samples were de-identified and otherwise considered medical waste. The tissue collection was approved as non-human subject (45 CFR 46.102(f)), which does not require consent, by the University of Southern California Health Sciences institutional review board (IRB: HS-11-00206). Only those from healthy mothers undergoing uncomplicated elective caesarean deliveries were utilized. Any patient testing positive for human immunodeficiency virus, hepatitis B virus, hepatitis C virus, tuberculosis, chlamydia trachomatis, neisseria gonorrhoeae, syphilis or any placenta showing any macroscopic abnormality were excluded. hAECs were enzymatically isolated from the amniotic membrane as previously described [26].

In brief, the amnion layer was mechanically separated from the chorion layer and washed several times with phosphate-buffered saline without calcium and magnesium (PBS) to remove blood. To dissociate AE cells, the amniotic membrane was incubated at 37 °C with 0.05 % trypsin containing 0.53 mM EDTA4Na (Life Technologies, Grand Island, NY, USA). Digesta from the first 10 minutes of the trypsin digestion were discarded to exclude debris. Cells from the second and third 40-minute digests were pooled and washed three times with PBS. The viability of the AE cells was determined by exclusion of trypan blue dye and counted with a hemocytometer. Cells were cultured at 65 × 10^3^/cm^2^ in high-glucose DMEM with 10 % FBS supplemented 10 ng/ml recombinant human epidermal growth factor (AF-100-15, PEPROTECH, Rocky Hill, NJ, USA). Unattached cells and debris were removed after 24 h. One million cells were used immediately for characterization as a day-0 sample. After culturing for 5 days, cells were dissociated with trypsin and cryopreserved as described below.

### Cryopreservation and thawing procedures

Five commercial xeno-free cryopreservation media were examined: CryoStor CS10, CryoStor CS5 (BioLife Solutions, Bothell, WA, USA), STEM-CELLBANKER (amsbio, Cambridge, MA, USA), CryoStem (Stemgent, Lexington, MA, USA), and Synth-a-Freeze (Life Technologies) (Table 1). Standard cryomedium (FBS-10: 90 % FBS + 10 % dimethyl sulfoxide (DMSO)) was used as a control. Cells were washed twice with PBS and resuspended at a concentration of 1 × 10^6^ cells/ml in each ice-cooled cryomedium, and transferred to cryotube vials (Nunc Cryotube, Thermo Scientific, Waltham, MA, USA) samples were cooled in a standard freezing container (Nalgene, Thermo Scientific) to −80 °C at −1 °C/min. After 24 h, samples were transferred to a liquid nitrogen tank and cryopreserved for 2 weeks at the vapor phase of liquid nitrogen (−151 to approximately −172 °C). To thaw cryopreserved samples, cryotubes were placed in a 37 °C water bath for rapid thawing until almost no ice was detectable. Cell viability was examined immediately after thawing, using the trypan blue exclusion method. Cells were washed once with culture medium and cultured in triplicate on 6-well culture plates, 4-chamber slides for chemifluorescence analyses, or 96-well plates for (3-(4,5-dimethyl-2-thiazolyl)-2,5-diphenyl-2H-tetrazolium bromide (MTT) assay at 37 °C, 5 % CO_2_ incubator.

**Table 1:** Tested cryopreservation media

Medium	Brand name	Provider	Cat number
1	CryoStor CS10	BioLife Solutions	210102
2	CryoStor CS5	BioLife Solutions	205102
3	STEM-CELLBANKER	amsbio	11897
4	CryoStem	Stemgent	01-0013-51
5	Synth-a-Freeze	Life Technologies	A12542-01

Commercially available xeno-free cryopreservation media used for this study

### Determination of cell viability and recovery

The impact of cryomedia on the cell repopulation cell viability was tested by MTT assay. Briefly, the AE cells (10^4^ cells/well) were plated into flat bottom 96-well tissue culture plates. After 48 h, 10 ul MTT solution (Biotium, Hayward, CA, USA) was added to 100 ul of medium in each well. The plates were incubated at 37 °C for 2 h. The 570 nm absorbance and 630 nm background signal was measured and subsequently subtracted from the signal absorbance to calculate a normalized absorbance value. Cell repopulation was defined by the MTT assay as the amount of viable cells repopulated following cryopreservation, and cell recovery was defined as the percentage of viable cells following cryopreservation as compared to the number of pre-freeze viable cells.

### Chemifluorescence analysis

To examine the effects of freezing on mitochondrial membrane stability, MitoTracker Red CMXRos (Life Technologies) was used to visualize the mitochondria of hAECs following the manufacturer’s instructions. Briefly, after thawing, hAECs were cultured on a permanox 4-chamber slide (Lab-Tek, Thermo Fisher Scientific) for 2 days. When cultures reached 50 % confluency, hAECs were permeabilized by immersion in 0.1 % Triton X-100 in PBS for 10 minutes and incubated in AE media containing 2 nM of Mitotracker Red CMXRos for 25 minutes. Cells were then washed twice with PBS and fixed in 2 % (w/v) paraformaldehyde in PBS. To examine the cell membrane and cytoskeleton integrity, cells were simultaneously stained with Alexa Fluor 488 conjugated phalloidin (1:40, Life Technologies). 4′,6-diamidino-2-phenylindole (DAPI) containing mountant (ProLong Gold Antifade Mountant, Life Technologies) was used to seal the slides and counterstain nuclei. The stained hAEC images were captured using either a LSM 5 Exciter confocal laser-scanning microscope or an Axiovert 200 fluorescence/live cell imaging microscope (ZEISS, Thornwood, NY, USA). The CMXRos fluorescence intensity was analyzed with a scientific image-analysis program, ImageJ. The chemifluorescent images were split to three RGB channels, with the blue channel images used to count the number of nuclei and the red channel images for quantification of the integrated density of CMXRos.

### Real-time quantitative PCR

Total RNA was isolated utilizing the RNeasy Plus Mini Kit (Qiagen, Valencia, CA, USA) following the manufacturer’s protocol and the cDNA was synthesized from 1 μg RNA using qScript™ cDNA SuperMix (Quanta Biosciences, Gaithersburg, MD, USA). Quantitative real-time PCR (qRT-PCR) was carried out using the ViiA™ 7 Real-Time PCR System (Life Technologies) with PerfeCTa SYBR Green SuperMix (Low ROX) master mix (Quanta Biosciences). All reactions were carried out on the same plate in duplicate with amplification of the target genes: POU domain class 5 transcription factor 1 (*OCT4*), SRY (sex determining region Y)-box 2 (*SOX2*), Nanog homeobox (*NANOG*), alpha-L-iduronidase (*IDUA*), branched chain keto acid dehydrogenase E1, alpha polypeptide (*BCKDHa*), and the endogenous housekeeping gene, peptidylprolyl isomerase A (*PPIA*). Relative quantitative evaluation of target genes was determined by comparative cycle threshold (Ct) method (2–(delta)(delta)Ct method). The delta-Ct value from the FBS-10 group was used as the biological reference value. Primer sequences were *hOCT4A*-F 5′-ctt cgc aag ccc tca ttt c-3′, *hOCT4A*-R 5′-gag aag gcg aaa tcc gaa g-3′, *Sox2*-F 5′-tgc tgc ctc ttt aag act agg ac-3′, *Sox2*-R 5′-cct ggg gct caa act tct ct-3′, *NANOG*-F 5′-aga tgc ctc aca cgg aga ct-3′, *NANOG*-R 5′-ttt gcg aca ctc ttc tct gc-3′, *IDUA*-F 5′-tgg agg agc aca ggc ttc-3′, *IDUA*-R 5′-cca gct gag gac gta ctg gt-3′, *BCKDHa*-F 5′- gac ctg gtg ttt ggc cag ta-3′, *BCKDHa*-R 5′- tgg gcc atg aat agt tcc ag-3′, *PPIA*-F 5′-cct aaa gca tac ggg tcc tg-3′, *PPIA*-R 5′-ttt cac ttt gcc aaa cac ca-3′.

### Flow cytometric analysis

Samples were prepared with 1 × 10^6^ cells in 100 ul PBS with 1 % FBS and incubated for 40 minutes at 4 °C with the following antibodies; fluorescein isothiocyanate (FITC) conjugated Anti-TRA-1-60 antibody (mouse IgM, FCMAB115F, Millipore, Billerica, MA, USA), FITC conjugated Anti-Human Embryonic Stem Cell Antigen-1 (HESCA-1) antibody (mouse IgM, FCMAB111F, Millipore) and FITC conjugated isotype mouse IgM (clone:11E10, eBioscience, San Diego, CA, USA). After incubation, cells were washed and analyzed with the BD LSR II Analyzer and BD FACSDiVaTM Software Version 6.1 (BD Biosciences, San Jose, CA, USA), or FlowJo 10.0.8 data analysis software (FLOWJO LLC, Ashland, OR, USA).

### Senescence detection assay

Cryopreserved hAECs were tested for senescence with the use of the cellular senescence detection kit (Biovision, Mountain View, CA, USA), which histochemically detects senescence-associated lysosomal β-galactosidase (SA-β-Gal) expression. After 2 weeks of cryopreservation in cryopreservation media, hAEC were maintained in the standard medium at 37 °C in 5 % CO_2_ for 21 days with three passages. Prior to the senescence detection assay, cells were seeded in 12-well plates at a density of 10^5^ cells/well. The hAECs were subsequently fixed and stained for 18 h at 37 °C according to manufacturer’s instructions. Five phase contrast images were randomly acquired from one well at × 10 magnification. The number of SA-β-Gal positive cells were counted and the ratio of senescent cells/total number of nuclear was calculated.

### Statistical analysis

Results are expressed as mean ± standard error of the mean (SEM). To minimize the influence of the variability of clinical samples, we normalized the data with FBS-10 data for each case and expressed as relative values. Statistical analysis was performed with Prism 5.0a (GraphPad Software, San Diego, CA, USA). Experimental and control groups were compared with paired or unpaired one-way analysis of variance (ANOVA) with Bonferroni post hoc analysis and Dunnett’s multiple comparison test. A value of *p* <0.05 was considered statistically significant.

## Results

### Impact of serum and xeno-free cryopreservation media on human amniotic epithelial cells

**Fig. 1:**
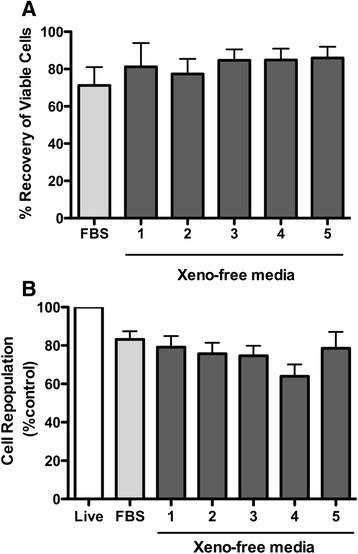
Comparison of cell recovery and repopulation capability. Cell viability was analyzed immediately after thawing, using the trypan blue exclusion method (n = 12). The mean value with standard error of the mean (SEM) of each group is presented (**a**). Cell repopulation capability after cryopreservation was evaluated 48 h after thawing, using the MTT assay (n = 12). The absorbance (A570–A630) values were normalized to unfrozen (*Live*) sample values. The percentage ratio with SEM of each group is presented (**b**)

A total of 18 human placentae were obtained to isolate hAECs. Two of them were excluded from the study due to the low cell attachment at the time of initial plating. In the remaining 16 cases, more than 70 % of isolated the hAECs attached to uncoated cell culture-grade dishes and demonstrated the typical cobblestone shape morphology under epidermal growth factor (EGF) supplementation as described previously [5]. The hAECs proliferated and reached about 80 % confluence on day 5 after isolation. Five commercial xeno-free cryomedia, proposed for stem cell cryopreservation, were selected; CryoStor CS10, CryoStor CS5 (BioLife), STEM-CELLBANKER (amsbio), CryoStem (Stemgent), and Synth-a-Freeze (Life Technologies) and were compared with a standard cryomedium (FBS-10: 90 % FBS + 10 % DMSO). All of these cryomedia contain 5 to approximately 15 % DMSO. The impacts of each xeno-free cryopreservation medium on post-thaw cell recovery and cell repopulation were evaluated (n = 12). The absolute number of viable cells in each tube was directly counted after cryopreservation by the trypan blue exclusion method utilizing a hemocytometer (Fig. 1a). The cell repopulation capability was evaluated 48 h after thawing by using a quantitative colorimetric MTT assay (Fig. 1b). After cryopreservation, no significant differences were observed in either cell viability or cell repopulation capability between the different cryopreservation media.

### Confocal microscopic analysis of cell membrane integrity and mitochondria damage after cryopreservation

**Fig. 2:**
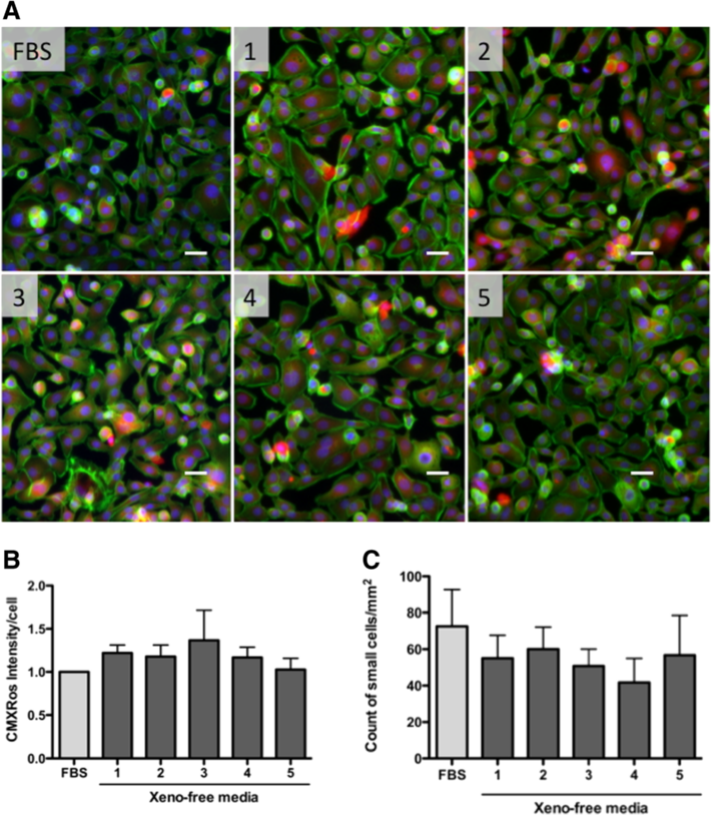
Cell and mitochondria morphology. Cell and mitochondria morphology was evaluated by chemifluorescence 2 days after thawing (n=8). The cells were stained with Alexa Fluor 488-phalloidin (*green*) to visualize actin filaments and MitoTracker Red (LifeTech, M7512) to evaluate mitochondria membrane stability (**a**). We used 4′,6-diamidino-2-phenylindole to counterstain the cell nuclei (*blue*). *Scale bars* represent 20 μm. CMXRos fluorescence intensity was measured with ImageJ and the average and standard error of the mean (SEM) were plotted as relative to the value of FBS control group (**b**). Numbers of small round cells were counted with ImageJ software and mean number of cells per mm^2^ of each group is presented with SEM (**c**).

Confocal microscopic fluorescent images illustrate the actin cytoskeleton and mitochondria quality after cryopreservation with each tested medium (Fig. 2a). Cells isolated from eight different placentae were examined. Two days after thawing, cells were stained with Mitotracker Red Chloromethyl-X-rosamine (CMXRos) and counterstained with Alexa488 conjugated phalloidin and DAPI. CMXRos is a lipophilic cationic fluorescent dye that is concentrated inside mitochondria. As this localization is dependent on the mitochondrial membrane potential, the fluorescence intensity represents the overall mitochondrial membrane potential of the cells. We have evaluated the CMXRos fluorescence intensity in each cryopreservation media exposed sample using a scientific image-analysis program, ImageJ (n = 8). The chemifluorescent images were split into three RGB channels; blue channel images were used to count the number of nuclei and red channel images were used to measure the integrated density of CMXRos. Each integrated density value was normalized by the number of nuclei to obtain an arbitrary number of CMXRos intensity per cell. The average and SEM values were plotted as relative to the value of FBS standard cryopreserved samples (Fig. 2b). CMXRos fluorescence intensity per cell was slightly higher in the CELL BANKER-treated cells, however these differences were not statistically significantindicating that all tested cryomedia preserved the mitochondrial membrane potential of these cells. There was no observable difference in cytoplasmic loss or mitochondrial clustering in any of the groups. Most cells revealed typical epithelial cell morphology on the uncoated permanox surface within a size of up to 30 μm. Some cells were small and round in shape (about 10 μm in diameter). Based on previously reported observations the small cells were expected to be undifferentiated or immature cells, which may retain stem cell-like characteristics [5]. There were no significant differences between the samples in the number of small cell populations (Fig. 2c).

### Stem cell characteristics of hAEC after preservation

**Fig. 3:**
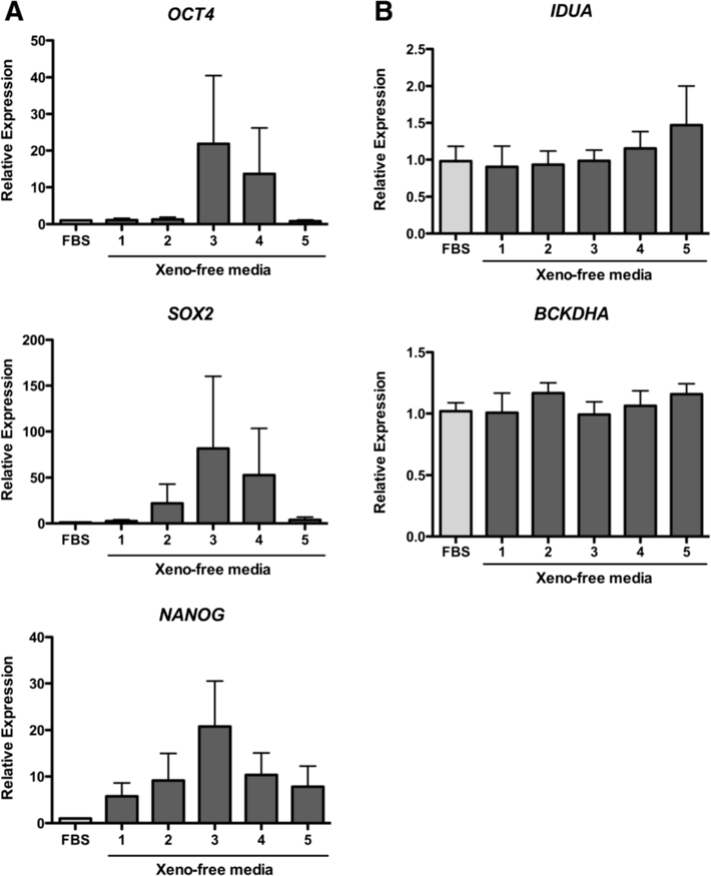
Stem cell marker genes expression. Real-time quantitative PCR analyses reveal relative stem cell marker gene (**a**) and functional enzyme gene (**b**) expression after cryopreservation (n = 12). Levels of gene expression were plotted against conventional FBS/10 % dimethyl sulfoxide (DMSO) cryopreservation medium

The expression of the stem cell marker genes (n = 12; *OCT4, SOX2* and *NANOG*) and the stem cell surface markers (n = 6; TRA1-60 and HESCA-1) was examined after cryopreservation. Cells cryopreserved with STEM-CELLBANKER best preserved expression of the stem cell marker genes (Fig. 3a). Significant differences were not detected between groups using one-way ANOVA; *OCT4* (*p* = 0.4513), *SOX2* (*p* = 0.6686), and *NANOG* (*p* = 0.2088). However, the relative expression of all stem cell marker genes in cells cryopreserved in STEM-CELLBANKER and CryoStem were increased as compared to the other three cryomedia. No differences were detected in expression of the functional enzyme genes, *IDUA* and *BCKDHa*, in cells preserved in the different media (Fig. 3b).

**Fig. 4:**
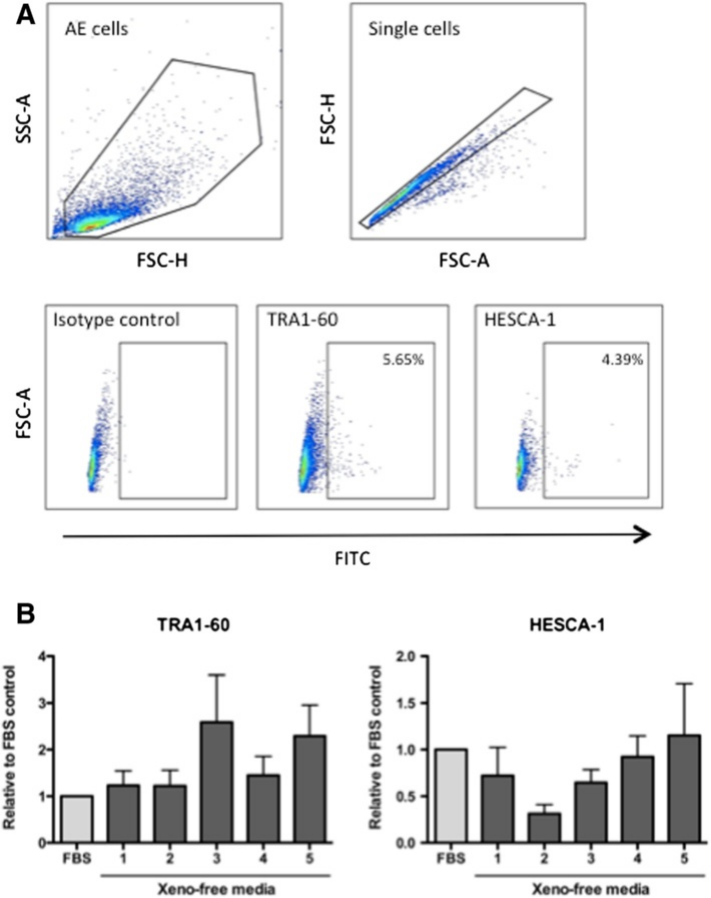
Stem cell surface marker expression. Representative pseudo-color density plots demonstrate gating strategy in flow cytometric analyses (**a**). The relative value of tumor rejection antigen (*TRA*) 1–60 and human embryonic stem cell antigen-1 (*HESCA-1*)-positive cells to control samples was plotted for the comparison of each xeno-free cryopreservation medium (**b**). *AE* amniotic epithelial, *SSC* side scatter, *FSC* forward scatter

Flow cytometric analyses were performed to examine the embryonic stem cell surface markers, TRA1-60 and HESCA-1 (n = 6). The forward scatter (FSC) and side scatter (SSC) gating revealed the hAEC to be a single cell population in cell size and complexity. Subsequently, the FSC-H and FSC-A gating was applied to identify single cell events. The ratio of stem cell surface marker positive cells was determined with the FITC intensity and FSC-A gating (Fig. 4a). Overall both stem cell markers were expressed in approximately 5 % of cryopreserved hAEC, however, this expression was varied in each placenta. In order to demonstrate the impact of different cryopreservation media, the relative value of each group as compared to FBS samples was calculated and analyzed with one-way ANOVA using Dunnet’s multiple comparison test (n = 6). Although STEM-CELL BANKER and Synth-a Freeze cryomedia relatively preserved more tumor rejection antigen (TRA)1–60-positive cells than other cryomedia, these differences were not significant (*p* = 0.2596). Nor was there a difference between the groups in the number of human embryonic stem cell antigen-1 (HESCA-1)-positive cells (*p* = 0.3680) (Fig. 4b).

**Fig. 5:**
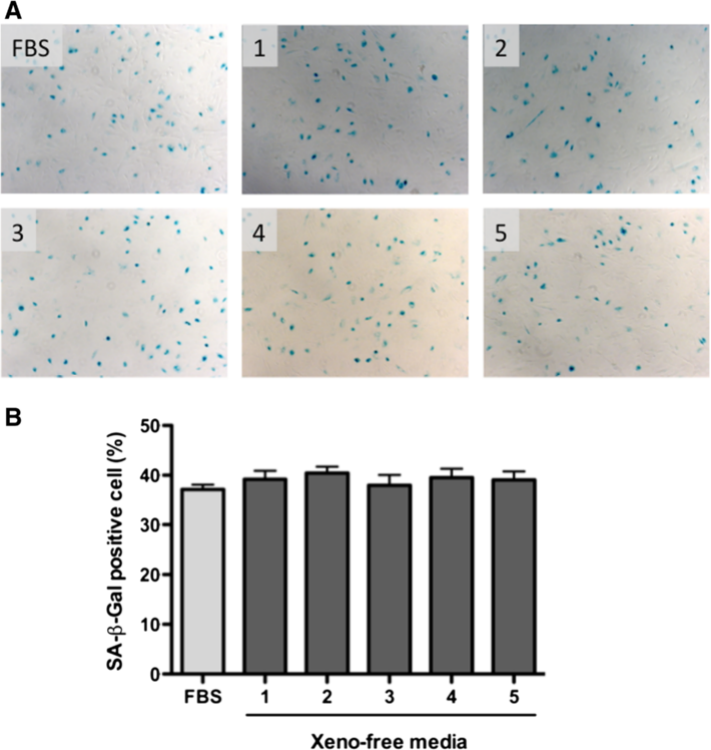
Post-cryopreservation senescence marker activity. Cryopreserved human amniotic epithelial cells were cultured for 21 days and the influence of cryopreservation was evaluated by senescence-associated-beta-galactosidase (SA-βgal) activity, which is detectable in the cytoplasmic blue color staining (**a**). The ratio of SA-β-Gal-positive cells was calculated and demonstrated as mean ± standard error of the mean (**b**)

### Senescence-associated lysosomal β-galactosidase activity in crypreserved hAECs

The ratio of senescence-associated lysosomal β-galactosidase (SA-β-Gal)-positive cells was not significantly different between the tested cryomedia (*p* = 0.7677, one-way ANOVA). In the FBS/DMSO control group 37.15 ± 3.601 % of hAEC were positive for SA-β-Gal, whereas other groups with xeno-free media preserved the following results were observed; CryoStor CS10: 47.22 ± 6.497 %, CryoStor CS5: 49.18 ± 5.081 %, STEM-CELL BANKER: 49.15 ± 8.118 %, CryoStem: 50.00 ± 6.951 %, and Synth-a-Freeze: 48.11 ± 6.588 % (Fig. 5). Consistent with the cell recovery and repopulation capability data, no impact of xeno-free cryomedia was observed and there was no significant difference between the tested xeno-free cryomedia.

## Discussion

The establishment of an efficient and simple cryopreservation protocol is crucial for a consistent supply of a sufficient number of cells for future clinical applications, as human pluripotent stem cells are known to be highly susceptible to freeze-thaw damage. Vitrification techniques have been used to improve the recovery of stem cells; however, the procedure is cumbersome and the high osmolality of the cryopreservation media are highly toxic to the cells. In this current study we have successfully applied commercially available serum-free and xeno-free chemically defined media to cryopreserve hAECs with a simple slow freezing protocol. Conventional slow freezing media contain 5–20 % DMSO and/or glycerol as cryoprotectants to prevent formation of micro ice crystals, which impair cytoplasmic structures including the cytoskeleton fibers [27, 28] and the mitochondrial membrane [29–32]. Traditional freezing medium also contains high concentrations of FBS, which increases not only the risks of undesired immunological reactions but also zoonosis. Over the last decade alternative cryoprotective substances, which avoid these potential problems have been developed, including disaccharide trehalose [33], oligosaccharide [34], and sericin [35]. These allow for the cryopreservation and recovery of human pluripotent stem cells without the loss of stem cell characteristics [36]. Among these newly developed cryopreservation media, we tested five different commercially available xeno-free chemically defined freezing media with a simple in-suspension slow freezing protocol. All five freezing media successfully cryopreserved human AE cells, and allowed for their recovery. We were able to visualize the linear polymer microfilaments, filamentous actin (F-actin), with fluorescein-conjugated phalloidin, and no differences were seen in the cytoskeletal morphology between hAECs preserved in the different media. Similarly, following cell staining with Mitotracker Red CMWRos, no differences were seen between the cryomedia in the effect on mitochondrial membrane stability.

Differences were observed between tested media in the preservation of both stem cell surface antigens and marker genes. Of the commercially available cryopreservation media tested, STEM CELL BANKER, appeared superior in preserving the pluripotent stem cell marker genes; *OCT4, SOX2* and *NANOG*, and the stem cell surface markers TRA1–60, however, this difference was not statistically significant. Our study further supports existing data showing the high cryopreservation efficiency of STEM CELL BANKER cryomedia, previously demonstrated in mouse induced pluripotent stem (iPS) cells [17], human iPS cells [37, 38], mesenchymal stem cells [39], and primary hepatocytes [40]. None of these studies compared these media with other commercially available xeno-free chemically defined freezing media or examined the effect of these media on transcription and expression of stem cell markers.

To the best of our knowledge, this is the first report to demonstrate that STEM CELL BANKER cryomedia preserves stem cell populations of primary hAECs and, when compared to other commercially available media, allows for improved maintenance of stem cell characteristics. Scanning electron microscopy would be useful in order to further analyze membrane integrity and structural alterations. It is unlikely however, that the impact on membrane integrity and structural alterations would influence only stem cell characteristics and not cell recovery and viability. It has been disclosed that STEM CELLBANKER contains 10 % DMSO, glucose, and high molecular weight polymer in PBS [37]. Due to the proprietary information on the exact contents of the media, further mechanism analysis on the preservation of stem cell characteristics is limited.

Two major advantages of using commercially available cryopreservation media are the availability and the quality. Both parameters are essential to establish a standardizing protocol, which can be applied to isolate hAECs in a wide range of otherwise non-standard conditions. Unlike other stem cells, amnion-derived stem cells can be isolated from human placentae, which are normally discarded as medical waste and therefore readily available in most countries. To establish an efficient biobank system, networking the cell supply with the standardized isolation/cryopreservation protocol is essential. A quality-controlled product is currently most suitable for this purpose. As mentioned above the STEM CELLBANKER media has already been utilized with various human cell types currently in consideration for clinical applications and thus, there are accumulating safety data for its use in clinical trials. In addition to providing consistent materials for use in any standardized protocol, this will allow for a more streamlined clearance of regulatory hurdles for its use with alternative cell sources. Taken together, commercially available cryopreservation media are good candidates for further investigation when establishing standardized cryopreservation protocols.

## Conclusions

In conclusion, the current study demonstrates that all tested commercially available xeno-free cryopreservation media successfully cryopreserved human amniotic epithelial cells without losing post-thaw cell viability and repopulation ability. Variable results were observed between the tested cryopreservation media in the preservation of stem cell characteristics of human amnion-derived stem cells. Although STEM CELLBANKER appeared to best maintain the stem cell markers in hAECs, the differences were not statistically significant. A larger sample size might better discern this. These data indicate that establishing a standardized cryopreservation protocol using commercially available xeno-free media is feasible for amnion-derived stem cell banking.

## Abbreviations

AE: amniotic epithelial
ANOVA: analysis of variance
BCKDHa: branched chain keto acid dehydrogenase E1, alpha polypeptide
Ct: cycle threshold
DAPI: 4′,6-diamidino-2-phenylindole
DMEM: Dulbecco’s modified Eagle’s medium
DMSO: dimethyl sulfoxide
ES: embryonic stem
FBS: fetal bovine serum
FITC: fluorescein isothiocyanate
FSC: forward scatter
GMP: good manufacturing practice
hAEC: human amniotic epithelial cell
IDUA: alpha-L-iduronidase
iPS: induced pluripotent stem
MTT: (3-(4,5-dimethyl-2-thiazolyl)-2,5-diphenyl-2H-tetrazolium bromide
NANOG: nanog homeobox
OCT-4: POU domain, class 5, transcription factor 1
PBS: phosphate-buffered saline
PPIA: peptidylprolyl isomerase A
SA-β-Gal: senescence-associated lysosomal β-galactosidase
SEM: standard error of the mean
SOX-2: SRY- sex determining region Y-box 2
SSC: side scatter
SSEA: stage-specific embryonic antigen
TRA: tumor rejection antigen
